# METABOLIC SYNDROME AND POOR SELF-RATED HEALTH AS RISK FACTORS FOR PREMATURE EMPLOYMENT EXIT

**DOI:** 10.1093/geroni/igad104.1489

**Published:** 2023-12-21

## Abstract

Poor self-rated health (SRH) is a well-established risk factor for premature employment exit through unemployment, work disability, and early retirement. However, it is not clear whether the premature employment exit risk associated with underlying cardio-metabolic health conditions is fully captured by poor SRH. This study examines the metabolic syndrome (MetS), an early-stage risk factor for cardiovascular disease and type two diabetes mellitus, as a risk factor for premature employment exit while controlling for poor SRH. We analyzed data from N=54,108 middle-aged and older Dutch workers (40-64 years) from five waves of the Lifelines Cohort Study and Biobank. MetS components were based on physical measures, blood markers, and medication use. SRH and employment states were self-reported. The associations between MetS, SRH, and premature employment exit types were analyzed using competing risk regression analysis. During follow-up (4.3 years), MetS was identified as an independent risk factor for unemployment (adjusted subdistribution hazard ratio (SHR): 1.13, 95% CI: 1.03, 1.25) and work disability (adjusted SHR: 1.35, 95% CI: 1.13, 1.61) when adjusted for poor SRH, chronic diseases, and sociodemographic factors. MetS was not associated with early retirement. To conclude, poor SRH did not fully capture the risk for unemployment and work disability associated with MetS. Workers with MetS might thus not be aware of their cardio- metabolic health related risks for premature employment exit. More awareness about MetS as a risk factor for premature employment exit is needed among workers, employers, and occupational health professionals. Further, MetS prevention might help to prolong working lives.

